# Elevated troponin I levels on admission predict long-term mortality in patients with acute cerebral infarction following thrombolysis

**DOI:** 10.1007/s10072-022-06116-6

**Published:** 2022-05-17

**Authors:** Yuehong Huang, Yanqi Shao, Yiqi Wang, Tianming Shi

**Affiliations:** 1 Department of Neurology, Hangzhou Mingzhou Hospital, Second Affiliated Hospital of Zhejiang University International Medical Center, 590 Shixin North Road, Zhejiang 310000 Hangzhou, China; 2 Center for Rehabilitation Medicine, Department of Neurology, Zhejiang Provincial People’s Hospital (Affiliated People’s Hospital, Hangzhou Medical College), Zhejiang 310014 Hangzhou, China

**Keywords:** Troponin I, Cerebral infarction, Prognosis, Thrombolytic therapy

## Abstract

**Background/objective:**

Cardiac diseases are frequently accompanied by elevated levels of biomarkers, among which, troponin is commonly investigated. The levels of plasma cardiac troponin I (cTnI), which has been shown to predict short-term mortality, are elevated in patients with acute cerebral infarction (ACI). However, few studies have assessed the association between cTnI concentration and long-term mortality in patients with ACI following thrombolysis.

**Methods:**

Patients with ACI admitted between January 1, 2014, and December 31, 2016, were registered. Data on demographics and outcomes with elevated cTnI levels were also collected.

**Results:**

A total of 145 patients with ACI were recruited; 97 (66%), 30 (20%), and 18 (12%) patients had cTnI concentrations < 0.030 (group 1), 0.030–0.10 (group 2), and > 0.10 μg/L (group 3), respectively. cTnI elevation was associated with older age, atrial fibrillation, congestive heart failure, renal insufficiency, coronary artery disease, stroke severity (National Institutes of Health Stroke Scale score), and prior smoking history at admission. After adjusting for comorbidities and severity at 3 months after ACI, cTnI elevation on admission was significantly associated with ascending 5-year mortality (hazard ratio, 1.80; 95% confidence interval, 1.22–2.65).

**Conclusions:**

Even after adjusting for several possible confounders, cTnI elevation in patients with ACI treated with rt-PA was associated with a 1.80-fold increased risk of 5-year mortality.

## Introduction

Cardiac diseases are frequently accompanied by elevated cardiac troponin (cTn) concentration. Elevated concentrations of cTn, including cTnI and cTnT, have been observed in many non-cardiac diseases, such as acute cerebral infarction (ACI) [1], subarachnoid hemorrhage [2], chronic kidney disease [3], severe infection [4], pulmonary embolism [5], cancer [6], and other diseases [7]. Elevated cTn has a prognostic value for short- and long-term mortalities in acute ischemic diseases or subarachnoid hemorrhage cases [2, 8, 9]. Considering the close relationship between acute cardiac disease and ischemic stroke, the current American Heart Association/American Stroke Association guidelines recommend the assessment of blood cTn concentration in patients diagnosed with ACI [10]. Elevated cTnI concentrations are observed in patients with ACI [1], indicating ascending risks of short-term mortality [1, 9]. When referring to patients with ACI treated with thrombolytic therapy, there are relatively few studies concerning the association between cTnI concentration and long-term mortality [11].

The exact mechanism of cTnI elevation in ACI remains controversial [1, 8, 9]. Some authors have proposed that it occurs owing to concomitant cardiac disease [1, 8, 9] or that cTnI in ACI is a biomarker of unstable atherosclerotic plaques in the cardiac coronary blood circulation [12]. Other studies have suggested that cTnI elevation following cardiac injury may be caused by an abnormal sympathetic nervous system condition, in which cortical areas controlling autonomic functions are destroyed, which is followed by ACI (e.g., insular cortex) [13].

Patients and relatives can determine the potential outcomes when prognostic information for ACI is provided. At least 30% of all patients with ACI who were treated with recombinant tissue-type plasminogen activator (rt-PA) thrombolytic therapy by intravenous infusion were more likely to have limited or no disability based on assessment using the National Institutes of Health Stroke Scale (NIHSS) at 3 months [14]. The mortality rate at 3 months was 17% in the rt-PA group [14]. In patients without intravenous thrombolysis, the 5-year mortality rate after the first ischemic stroke was approximately 41% [15]. However, few studies have reported on the long-term mortality (> 5 years) owing to troponin elevation in patients with ACI following thrombolytic therapy. Therefore, the main purpose of this study was to describe the patient characteristics, long-term mortality, and frequency and determinants of elevated plasma cTnI levels in patients with ACI following thrombolysis.

## Methods

### Study population

We consecutively collected data from patients with ACI who registered at the stroke center of our hospital within 4.5 h of the stroke between January 1, 2014, and December 31, 2016. After undergoing computed tomography (CT) of the brain and CT angiography (CTA) of the carotid and cerebral areas, based on the patients’ consent and choices, they were provided with intravenous rt-PA thrombolytic therapy or thrombolytic therapy following endovascular therapy (ET).

### Inclusion and exclusion criterion

The inclusion criteria were as follows: (a) a diagnosis of ACI based on the International Classification of Diseases, 10th revision (code I63.x); (b) age > 18 years; (c) presence of ACI owing to vascular obstruction confirmed by carotid and cerebral CTA or cerebral digital subtraction angiography; (d) absence of intracranial hemorrhage on admission brain CT scan; and (e) ET initiation within 6 h of symptom onset if CTA revealed main vascular obstruction.

The exclusion criteria were as follows: (a) acute cardiac disease, comprising acute atrial fibrillation, acute heart failure, acute myocardial infarction, acute coronary syndrome, and pulmonary embolism; (b) cerebral hemorrhage; and (c) unsuitability for treatment with intravenous thrombolytic therapy.

### Intravenous thrombolysis and ET

All enrolled patients were treated with intravenous rt-PA thrombolytic therapy. Patients who underwent endovascular surgery were implanted with 8- or 6-Fr guiding catheters, which were placed in the common carotid or internal arteries through a femoral sheath under local anesthesia. Thrombectomy was performed in all patients using a Solitaire AB device (Solitaire, Covidien, Irvine, CA, USA).

### Clinical data collection

The clinical characteristics obtained by experienced neurologists included a previous history of cerebrovascular disease (CVD) (ischemic stroke or transient ischemic attacks), high blood pressure, atrial fibrillation (AF; previous history of AF or electrocardiogram obtained during the hospital stay), congestive heart failure (CHF), coronary artery disease (CAD), hyperlipidemia (HLP; previous history of HLP or triglyceride level > 1.69 mmol/L, low-density lipoprotein cholesterol > 3.1 mmol/L, or total cholesterol > 5.96 mmol/L on admission), type 2 diabetes mellitus, renal insufficiency (RI) (creatinine > 133 μmol/L on admission), smoking history, and prior pharmacotherapy (calcium channel blockers (CCBs), angiotensin receptor blockers (ARBs), angiotensin-converting enzyme inhibitors (ACEIs), beta-blockers, and lipid-lowering drugs).

The white blood cell (WBC) counts and cTnI concentrations were measured as early as possible upon presentation at the emergency department within 4.5 h of stroke onset. The second in-hospital blood cTnI measurement was performed at 24–48 h after ACI treated with intravenous thrombolysis, regardless if the initial cTnI concentration was above or below the 99th percentile of the normal values (> 0.1 μg/L). If the value of cTnI was > 0.01 μg/L, we continued to test until reaching the normal range (< 0.01 μg/L). If the index of cTnI continued to rise, we conducted relevant examinations to exclude heart disease. We also measured the blood sugar, blood lipid, and creatinine levels. The cTnI levels were measured with a two-site sandwich immunoassay using an ADVIA Centaur analyzer (AccuTnI+3; Beckman Coulter Inc., Brea, CA, USA). The recruited patients were divided according to their cTnI concentrations: normal (group 1; < 0.03 μg/L) and elevated groups (group 2 (> 0.03 and < 0.1 μg/L) and group 3 (> 0.1 μg/L)).

**Fig. 1:**
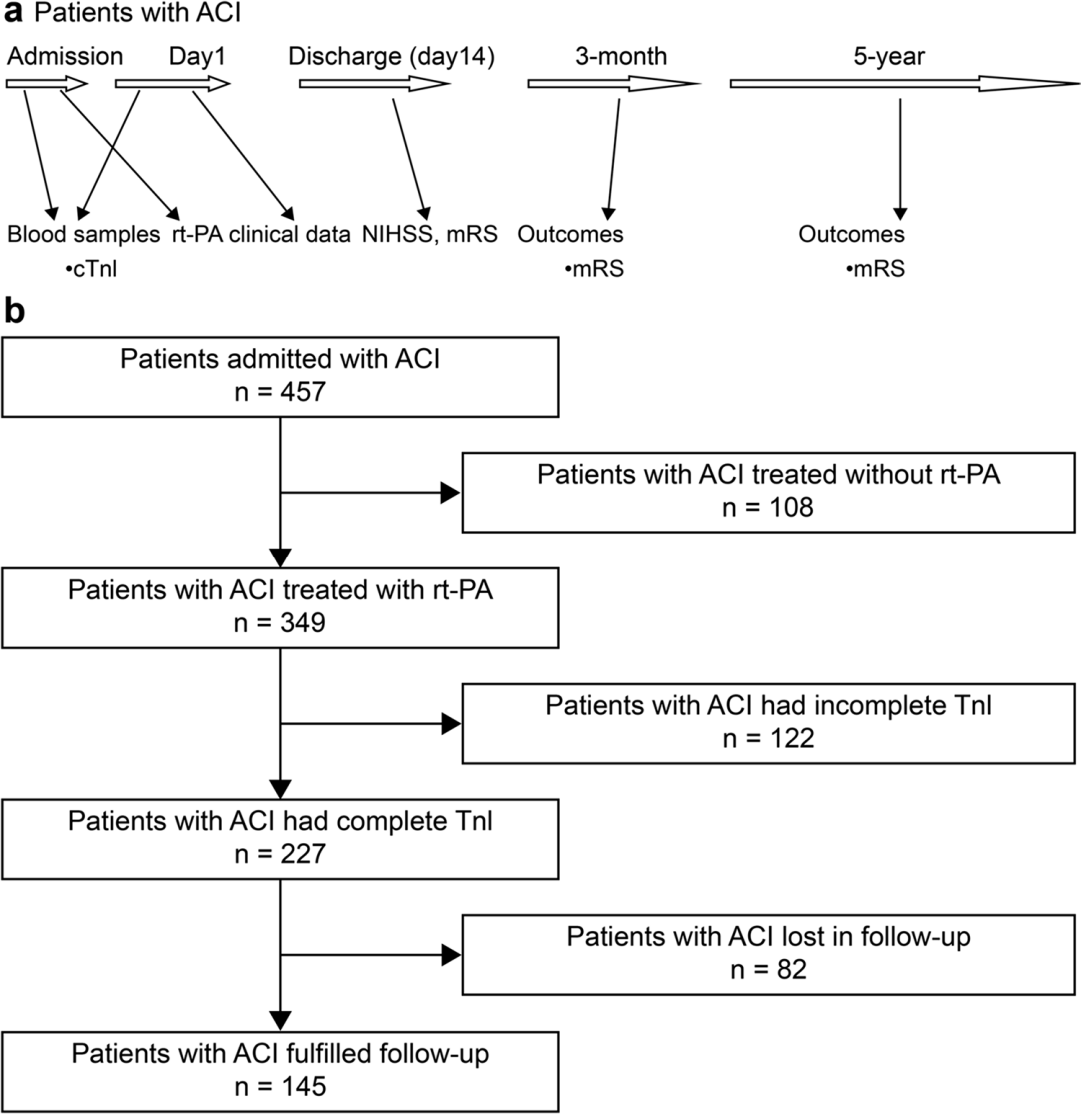
Study protocol and flow chat of the included study population. **a** Study protocol. Blood samples for cTnI were obtained on admission and on the following day. Clinical data were recorded during in-hospital stay. The NIHSS and mRS scores were evaluated on admission and discharge. Outcome was evaluated with the mRS score at 3 months and 5 years after admission. **b** Flow chart of the included study population. Abbreviations: ACI, acute cerebral infarction; cTnI, cardiac troponin I; mRS, modified Rankin Scale; NIHSS, National Institutes of Health Stroke Scale

Brain magnetic resonance imaging examination was performed upon admission to confirm the lesion location. ACI was classified according to the TOAST criteria [16]. The ACI severity was evaluated using the NIHSS [17] and modified Rankin Scale (mRS) scores, and functional prognosis was assessed using the mRS scores obtained via telephone calls after 3 months and 5 years. The clinical endpoints were all-cause death or the end date of May 29, 2021. For cases in which the patients could not respond to questions, their nearest relatives or primary care physicians were interviewed. The study protocol is shown briefly in Fig. 1a.

### Statistical analyses

Categorical data are presented as percentages. Continuous variables are expressed as means of the median values and standard deviations (SDs). The Mann–Whitney *U* tests were used to compare the differences between the normal (group 1) and elevated groups (groups 2 and 3). We used the Kruskal–Wallis *H* tests to assess the differences among the three groups.

We used the Kaplan–Meier curves to describe the survival time of the different levels of elevated cTnI. By eliminating the non-statistically significant covariates and reserving the correlated variables, multivariate Cox regression was used to analyze the variables that were significantly correlated with mortality.

The data obtained are expressed as hazard ratios (HRs) with corresponding 95% confidence intervals (CIs). *P*-values of < 0.05 were considered statistically significant. Statistical analyses were performed using the Statistical Package for the Social Sciences version (SPSS) 16.0 (SPSS Inc., Chicago, IL, USA). This study was approved by the Regional Committee for Ethics of local hospital.

## Results

### Demographics

A total of 457 patients, with onset-to-door time of 4.5 h, were consecutively registered as having cerebral infarction. Among them, 349 (76%) patients received intravenous rt-PA thrombolytic therapy, and the 108 remaining patients refused to receive thrombolytic therapy. Of the 349 patients, 122 did not have troponin results, while 82 had missing follow-up data and were excluded. Finally, this study included 145 patients with ACI who received intravenous rt-PA thrombolytic therapy. The flow chart is presented in Fig. 1b.

In this study, 97 (66%), 30 (20%), and 18 (12%) patients had cTnI concentration levels of < 0.030 (group 1), 0.030–0.10 (group 2), and > 0.10 μg/L (group 3), respectively. The demographic characteristics of each group are presented in Table 1.

**Table 1:** Demographics characteristics with normal and elevated cTnI concentration

	Group 1	Group 2	Group 3	Three groups^*b^	Group 1 vs. groups 2 and 3^*a^
cTnI (μg/L)	< 0.030	0.030 < cTnI < 0.10	> 0.10		
Clinical characteristics	(*n* = 97)	(*n* = 30)	(*n* = 18)		
Years (mean ± *SD*)	66.7 ± 12.2	73.9 ± 14	75.5 ± 9.3	0.001	0
Male (%)	60 (61.8)	16 (53.3)	13 (72.2)	0.425	0.867
*Medical history*					
Ischemic stroke (%)	61 (62.8)	20 (66.6)	15 (83.3)	0.244	0.231
Hypertension (%)	72 (74.2)	20 (66.6)	13 (72.2)	0.722	0.489
Congestive heart failure (%)	0	1 (3.3)	4 (22.2)	0	0.001
Coronary artery disease (%)	2 (2)	3 (10)	3 (16.6)	0.022	0.010
Atrial fibrillation (%)	21 (21.6)	16 (53.3)	11 (61.1)	0	0
Diabetes mellitus (%)	17 (17.5)	9 (30)	2 (11.1)	0.207	0.441
Smoking (%)	42 (43.2)	5 (16.6)	4 (22.2)	0.014	0.004
*Laboratory data*					
Hyperlipidemia (%)	23 (23.7)	4 (13.3)	3 (16.6)	0.429	0.203
Leukocyte count (mean ± *SD*)	8.0 ± 2.88	7.4 ± 2.49	10.3 ± 3.87	0.013	0.501
Renal insufficiency (%)	2 (2)	2 (6.6)	3 (16.6)	0.026	0.028
*Treatment on admission*					
Calcium antagonists (%)	29 (29.8)	9 (30)	5 (27.7)	0.904	0.928
ACEI/ARB (%)	16 (16.4)	5 (16.6)	2 (11.1)	0.841	0.768
Beta-blockers (%)	8 (8.2)	3 (10)	2 (11.1)	0.920	0.668
Statins (%)	61 (62.8)	16 (53.3)	13 (72.2)	0.412	0.774
Endovascular therapy (%)	14 (14.4)	4 (13.3)	2 (11.1)	0.929	0.752
NIHSS score (mean ± *SD*)	9.32 ± 9.02	14.46 ± 9.3	16.5 ± 9.3	0	0
Intracranial hemorrhage	8 (8.2)	3 (10)	1 (5.5)	0.865	0.986
Insular cortex involvement	37 (38.1)	15 (50)	8 (44.4)	0.497	0.263
Left insular lobe	20 (20.6)	7 (23.3)	2 (11.1)	0.573	0.792
Right insular lobe	17 (17.5)	8 (26.6)	5 (27.7)	0.410	0.183
*Stroke subtypes (TOAST)*				0.191	0.069
Large artery atherosclerosis	30 (30.9)	13 (43.3)	9 (50)		
Cardiogenic embolism	28 (28.8)	9 (30)	4 (22.2)		
Small vessel disease	19 (19.5)	5 (16.6)	1 (5.5)		
Undetermined etiology	19 (19.5)	3 (10)	4 (22.2)		
Other etiologies	1 (1)	0	0		

^a^Mann–Whitney *U* test. ^b^Kruskal–Wallis *H* test. ^*^p-value. Abbreviations: *ACEI*, angiotensin-converting enzyme inhibitor; *ARB*, angiotensin receptor blocker; *cTnI*, cardiac troponin I; *NIHSS*, National Institutes of Health Stroke Scale; *SD*, standard deviation

Twelve, two, and four patients had cTnI levels of 0.10–1.0, 0.1–5.0, and > 10 μg/L, respectively. The patients in the cTnI elevated groups were older than those in the normal group. Analysis of the estimated normal cTnI group (< 0.030 μg/L) compared with the elevated cTnI groups (> 0.030 μg/L) showed evidently higher ratios of AF, CHF, CAD, and smoking in patients with cTnI levels > 0.03 μg/L. The prevalence of RI was elevated in patients with cTnI concentrations > 0.03 μg/L. The WBC count was greater in group 3 (cTnI > 0.10 μg/L) than in groups 1 and 2, but the statistical difference was > 0.5 when the normal (group 1) and elevated groups (groups 2 and 3) were compared.

The rates of previous medication use (CCB, ACEIs, ARBs, beta-blockers, and lipid-lowering drugs) were not different between the groups. In the three groups, patients with elevated cTnI level showed no difference in stroke subtypes, although comparisons of normal and elevated cTnI levels (< 0.03 vs. > 0.03 μg/L) demonstrated a trend of differences between them (*p* = 0.067). We also did not find a significant difference among the three groups with respect to ACI location (left and right sides of the insular lobe). The NIHSS scores recorded at admission were significantly higher in the cTnI elevated groups (> 0.03 μg/L). The incidence of complications, such as cerebral hemorrhage, after thrombolysis was not different among the groups. Twenty patients were treated with ET. The percentage of patients who received ET treatment did not differ among the three groups.

### Prognostic data

During the 5-year follow-up period, 44 (31%) patients died of different causes. The longest follow-up period was 2676 days (7.3 years). The median follow-up time was 2088 days (*SD*,± 291; 5.7 years). The functional outcomes of the patients (assessed using the mRS) in the three groups are shown in Table 2.

**Table 2:** Patients’ functional outcomes (assessed using the mRS score) with normal and elevated cTnI levels

	Group 1	Group 2	Group 3	Three groups	Normal versus elevated cTnI level
cTnI (μg/L)	< 0.03	0.03 < cTnI < 0.10	cTnI > 0.1	*p-*value	*p-*value
In-patient	3.19 ± 1.47	4.1 ± 1.14	4.6 ± 0.97	0	0
Discharge	2.29 ± 1.7	3.2 ± 1.56	4.05 ± 1.78	0	0
3 months	1.94 ± 1.93	2.6 ± 2.17	4 ± 2.4	0.002	0.003
5 years	2.05 ± 2.36	4 ± 2.23	4.6 ± 2.45	0	0

Abbreviations: *cTnI*, cardiac troponin I; *mRS*, modified Rankin Scale

**Fig. 2:**
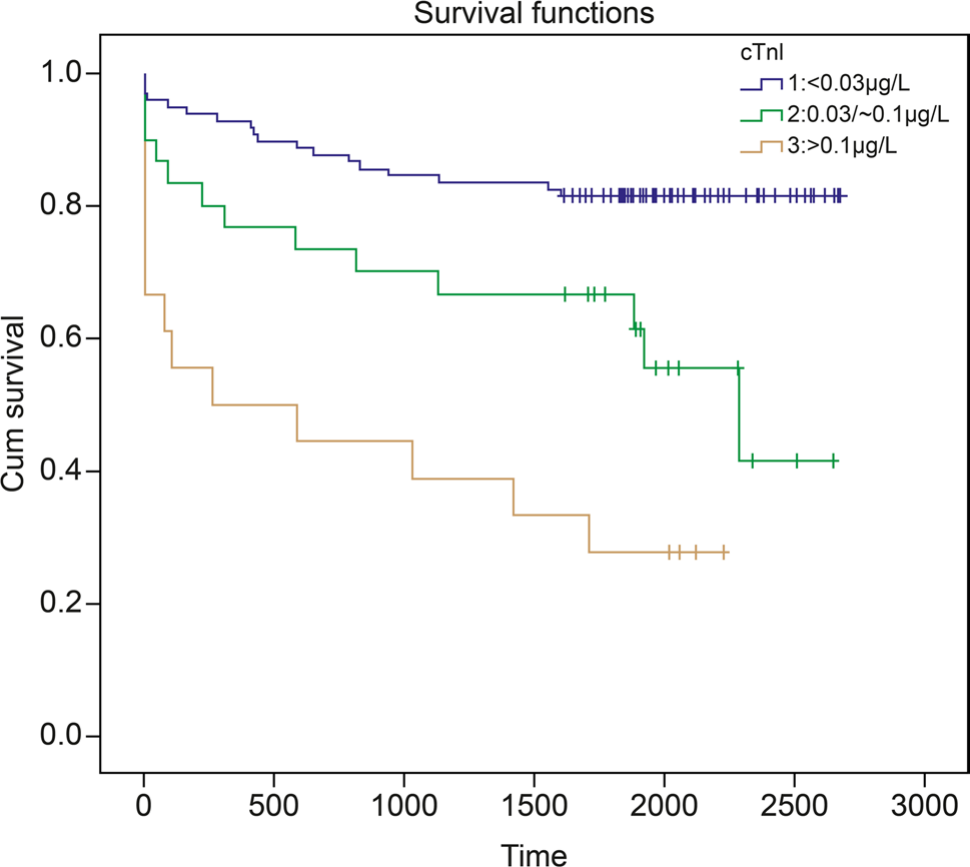
Long-term prognosis of cardiac troponin I elevation in patients with ACI. (1) Group 1 (cTnI < 0.03 μg/L); (2) group 2 (cTnI: 0.03–0.10 μg/L); and (3) group 3 (cTnI > 0.1 μg/L). The accumulated survival of thrombolysis-treated ACI patients stratified according to normal, low, and highly elevated cTnI levels. Kaplan–Meier curve (adjusted for ischemic stroke, atrial fibrillation, congestive heart failure, and mRS score at 3 months). Abbreviations: ACI, acute cerebral infarction; cTnI, cardiac troponin I; mRS, modified Rankin Scale

Overall mortality increased with increasing cTnI levels, and this risk factor persisted from the beginning to the end of the 5-year period (Fig. 2). Elevated cTnI levels were also associated with 3-month mortality (19/145, *p* = 0.005). Univariate analysis indicated that mortality was associated with age, right insular lobe location, stroke severity, CHF, AF, CVD, mRS score at 3 months, and elevated cTnI levels. After adjusting for CHF, AF, CVD, and mRS score at 3 months, a multivariate Cox survival analysis showed that the elevated cTnI group had significantly higher mortality rates than the normal group (cTnI < 0.03 μg/L, *HR*, 1.80; 95% *CI*, 1.2–2.65) *(*Table 3).

**Table 3:** Results of Cox regression analyses and parameters in all-cause mortality

	Univariate analysis		Multivariate analysis	
Clinical characteristics	*HR* (95% *CI*)	*p-*value	*HR* (95% *CI*)	*p-*value
Age	1.017 (1.003–1.032)	0.017	1.00 (0.98–1.03)	0.236
Ischemic stroke	0.242 (0.153–0.383)	0	0.21 (0.13-0.35)	0
Atrial fibrillation	1.56 (1.098–2.205)	0.013	1.55 (1.05–2.31)	0.027
Congestive heart failure	6.08 (2.37–15.54)	0	4.09 (1.43–11.67)	0.008
Endovascular therapy	2.15 (1.32–3.52)	0.002	1.11 (0.61–2.01)	0.723
Right insular lobe	1.64 (1.09–2.47)	0.017	1.16 (0.74–1.82)	0.506
cTnI > 0.030 μg/L	1.90 (1.33–2.70)	0	1.80 (1.22–2.65)	0.003
NIHSS score	1.02 (1.00–1.03)	0.021	1.0 (0.98–1.03)	0.463
mRS score—in-hospital	1.20 (1.06–1.36)	0.003	0.90 (0.743–1.10)	0.317
mRS score—discharge	1.15 (1.03–1.28)	0.008	0.83 (0.68–1.0)	0.058
mRS—3 months	1.26 (1.15–1.38)	0	1.37 (1.17–1.60)	0

Abbreviations: *CI*, confidence interval; *cTnI*, cardiac troponin I; *HR*, hazard ratio; *mRS*, modified Rankin Scale; *NIHSS*, National Institutes of Health Stroke Scale

No significant differences were found among the groups concerning age, cerebral hemorrhage after thrombolysis, or ACI subtype during the follow-up period.

## Discussion

In 2000, James first reported the relationship between mortality and cTnI elevation in patients with ACI [18]. Herein, we observed cTnI elevation in patients with ACI treated with rt-PA therapy. Despite adjusting for four possible confounders, cTnI was associated with a 1.8-fold increased risk of 5-year mortality. The cTnI level was also associated with the 3-month mortality rate. The ascending risk was maintained during the entire 5-year follow-up period. The ascending risk of mortality in patients with ACI treated with intravenous thrombolysis at admission is consistent with a previous study’s results on 3-month mortality [11]. The proportions of our patients with cTnI elevation and ACI treated with rt-PA therapy (33%) were similar to those reported previously (36%) [11]. We extended the observation time to approximately 5.7 years, whereas other studies assessed short-term or in-hospital outcomes [1, 9].

Elevation of cTnI level in the context of ACI in patients treated with rt-PA therapy was associated with older age and higher occurrence of CHF, CAD, AF, and RI. These findings are consistent with the following previously-reported data [1, 9]: (a) cTnI elevation has been reported in both acute and chronic patients with CHF, which may be attributed to the vulnerability of myocardial cells, chronic subclinical ischemia, or left ventricular insufficiency [8]; (b) RI was reported to be the cause of cTnI elevation [3]; (c) overall, 12.5% of the patients who first developed AF showed elevated cTnI concentration [19], which was explained by the fact that the heart experienced ischemia, with consequent cardiomyocyte necrosis. Both CHF and AF are strong predictors of mortality. Another study reported mild cTnI elevation in 830 stable patients with vascular disease, which included 491 and 341 patients with CAD and stroke, respectively [8]. We found that cTnI concentration > 0.03 μg/L independently predicted the all-cause 5-year mortality (*HR*, 1.76). Cardiovascular disease is considered the leading cause of long-term mortality in patients with stroke [20]. However, in our study, CAD was not significantly associated with mortality in the univariate or multivariate Cox regression models.

We observed a positive association between elevated blood cTnI concentrations and stroke severity (scored using the NIHSS) (*p* < 0.05), consistent with the result of a previous study [1]. Furthermore, the 3-month stroke severity (assessed using the mRS score) also significantly influenced mortality (odds ratio, 1.26; *p* < 0.001), consistent with the result of a previous study [11]. Jan et al. reported that elevated cTn levels were significantly associated with right insular cortex involvement [13]. A previous work reported that impairment of the insular lobe (right or left) increases with elevated cTn concentration [9]. However, Barber et al. did not identify a significant association between cTnI elevation and insular cortex stroke [21]. Similarly, we cannot make a conclusion concerning the association between elevated cTnI levels and the corresponding part of the affected brain artery territory (right or left insular cortex). Jan et al. reported that elevated cTn levels are more often observed in patients with acute cardioembolic stroke [13]. However, we did not find that the etiological classification of ACI differed between the normal and elevated cTnI levels.

Faiz et al. observed no significant correlation between rt-PA therapy and long-term mortality (558 ± 254 days) [22]. However, rt-PA therapy can improve the 3-month prognosis of ACI patients [14, 23]. The 3-month mortality rate in the present study was approximately 13%, slightly higher than that reported in a previous work (5%) [11], but similar to that reported in another study (17%) [14]. This contradiction is possibly attributed to our small sample size. The 5-year mortality rate in our patients with ACI was approximately 31%, which was significantly lower than that observed in patients with acute stroke with similar follow-up periods (40–60% fatality rate within 5 years after ACI) [24, 25]. This may be attributed to thrombolysis therapy and different patient characteristics. In our study, patients who died had a higher likelihood of undergoing ET (8/44, 18% vs. 12/101, 11%). However, we could not determine whether ET resulted in more deaths. ET was associated with considerable functional improvement in several randomized trials [25]. The higher mortality rate may be attributed to the obstruction of large intracranial vessels in patients with ACI, which would indicate that massive cerebral infarction is the main cause of death and can lead to increased mortality [27]. Cao et al. reported a 1-month mortality rate of approximately 36.2% in patients with troponin T high-sensitivity elevation who underwent ET [28], which was slightly higher than that observed in our study. However, ET did not increase the mortality rate, suggesting that the severity of disease based on the NIHSS score is more relevant than that in patients with ACI [20].

In this study, we present data that show a significantly higher WBC count in patients with cTnI elevation on admission, a finding consistent with that of a previous study [24]. These observations may result from stress that the patients feel when ACI occurs, thus resulting in increased WBC counts, simultaneous hyperexcitation of the sympathetic system, and release of several catecholamines, which may cause excessive contraction of cardiac muscle cells and, ultimately, increased cTn levels, particularly in patients with cardiac disease. This has also been demonstrated in stress-induced ischemia tests [29].

Many studies have reported the relationship between elevated cTnI concentration and the ischemic territory of cerebral infarction [30, 31], especially in the right part of the insular lobe. The insular lobe is part of the center of the autonomic nervous system, which is associated with the cerebral control of the sympathetic and parasympathetic outflow to the heart. An acute ischemic lesion in the insula results in the imbalanced control of the heart, which ultimately increases cTn concentrations through myocardial cell necrosis [32]. However, our study did not find a significant association between these factors.

The exact mechanism between elevated TnI and ACI is not clear. We found that the TnI level was elevated in ultra-early stage of disease. With retesting, estimation of TnI in many patients may increase or decrease, but none of them had sustained increase. One explanation of the condition is the cardiac stress reaction in patients with excessive release of catecholamine, resulting in the elevation of TnI [21]. Another explanation is that patients with elevated TnI level were accompanied with CAD or the presence of culprit plaques in the coronary arteries. Nevertheless, the troponin elevation in an acute ischemic stroke study reported that patients with elevated Tn in ACI underwent coronary angiography, suggesting that the coronary disease-causing plaque and stenosis in such patients were less frequent than those in patients with acute coronary syndrome without ST elevation [33]. Future studies are needed to record TnI concentration dynamically and cardiac examination (catecholamine, electrocardiograph, and coronary angiography).

This study has some limitations. First, all patients received intravenous rt-PA thrombolytic therapy. This is a unique population of patients with ACI. Second, a large number of patients were excluded because of missing cTnI values on admission or the loss of mRS data during follow-up, resulting in a small sample size. Third, we did not confirm heart disease by coronary CTA or coronary angiography, although we conducted electrocardiography and cardiac ultrasonography. In particular, we confirmed heart disease via clinical examinations. Nevertheless, the cardiologists did not establish definite diagnoses of acute myocardial infarction. Fourth, we did not measure the cTnI concentration continuously, although we determined the index of patients with cTnI concentrations > 0.1 μg/L until the levels were normalized. Unfortunately, we did not test all patients continuously. Fifth, our results were insufficient to demonstrate differences in the ACI subtypes. Scheitz et al. reported an evident contrast in the ratio of cardioembolic stroke between a cTnI elevated and a normal group [9]. Sixth, although new test systems, such as hypersensitive assays of troponin, are frequently used for detecting cardiovascular disease in patients with ACI, we used the conventional method to test cTnI. Future studies may use these newly developed troponin immunoassays to identify more cases of cardiovascular diseases. Moreover, we only detected the single biomarker, cTnI, other indexes, such as N-terminal prohormone of brain natriuretic peptide, copeptin, matrix metalloproteinase-9, and S100 β, are all independently associated with various clinical outcomes [34].

## Conclusions

Thrombolysis-treated patients with ACI who have elevated cTnI levels more often had serious strokes compared to those who had no cTnI elevation. These patients also had more concomitant conditions compared to those with normal cTnI levels. Furthermore, we confirmed the ascending risk of 5-year mortality in these patients with elevated cTnI levels on admission. Our data indicated the possibility that cTnI evaluation in these patients may be predictive of long-term mortality. More studies are needed to further clarify the mechanisms underlying elevated troponin levels in patients with ACI.

## Data Availability

The datasets generated during and/or analyzed during the current study are available from the corresponding author on reasonable request.
